# Effect of surgical intervention on the expression of leukemia inhibitory factor and L-selectin ligand in the endometrium of hydrosalpinx patients during the implantation window

**DOI:** 10.3892/etm.2012.728

**Published:** 2012-09-28

**Authors:** YIPING ZHONG, JIN LI, HAITAO WU, YING YING, YAFENG LIU, CANQUAN ZHOU, YANWEN XU, XIAOTING SHEN, QUAN QI

**Affiliations:** 1 Reproductive Medicine Center, The First Affiliated Hospital, Sun Yat-sen University, Guangzhou, Guangdong 510080;; 2 Guangdong No. 2 Provincial People’s Hospital, Guangzhou, Guangdong 510000, P.R. China

**Keywords:** hydrosalpinx, leukemia inhibitory factor, L-selectin ligand, implantation window, endometrial receptivity

## Abstract

The aim of this study was to investigate the effect of surgical intervention on the expression of leukemia inhibitory factor (LIF) and L-selectin ligand in the endometrium of patients with hydrosalpinx during the implantation window. A total of 60 patients with hydrosalpinx and 30 patients with tubal obstruction were recruited, and immunohistochemistry was performed to detect the expression of LIF and L-selectin ligand in the endometrium of hydrosalpinx patients before and after surgery and in the endometrium of patients with tubal obstruction. The expression of LIF and L-selectin ligand in the endometrium of hydrosalpinx patients before surgery was markedly lower than that of patients with tubal obstruction (P<0.05). Following surgery, the expression of LIF and L-selectin ligand in the endometrium of hydrosalpinx patients was comparable to that of patients with tubal obstruction (P>0.05). In addition, there was a pronounced difference in the expression of LIF and L-selectin ligand in the endometrium before and after surgery in patients with hydrosalpinx (P<0.05). Hydrosalpinx reduces the expression of LIF and L-selectin ligand in the endometrium during the implantation window. LIF and L-selectin ligand may be important factors influencing the endometrial receptivity of hydrosalpinx patients, and surgery is capable of improving the expression of LIF and L-selectin ligand in the endometrium during the implantation window.

## Introduction

The endometrium undergoes cyclic regeneration during the control of ovarian steroid hormones, and the blastocysts are only able to implant in the uterus in the appropriate phase (1). Blastocyst implantation is a shared phenomenon in mammalians. At approximately 3–6 days after fertilization (corresponding to D21–D24 in the natural cycle or D5–D8 in the luteinizing hormone peak) which is also known as the implantation window, the blastocysts enter the uterus, and then the blastocysts and the uterus secrete related proteins and local factors in a strict spatial and temporal order. These proteins and factors recognize each other and interact to assist the implantation (2). These biologically active cytokines and proteins are also known as markers for endometrial receptivity.

In females with infertility, tubal factors are the most common and account for 40% of the causes of female infertility. In addition, hydrosalpinx accounts for 10–30% of the causes of tubal factor infertility. Initially, *in vitro* fertilization-embryo transfer (IVF-ET) was used to treat infertility due to tubal factors. However, numerous studies have demonstrated that hydrosalpinx significantly reduces the implantation and pregnancy rate (3). The mechanism underlying the influence of hydrosalpinx on IVF-ET remains unclear. Previous studies have revealed that the influence of hydrosalpinx on the endometrial receptivity is one of these causes (4). In the present study, the expression of leukemia inhibitory factor (LIF) and L-selectin in the endometrium was compared between hydrosalpinx patients and tubal obstruction patients, and in hydrosalpinx patients before and after surgery during the implantation window. This study aimed to explore the causes of a poor outcome of IVF-ET in hydrosalpinx patients.

## Patients and methods

### Patients

A total of 60 patients with hydrosalpinx and 30 patients with tubal obstruction but without hydrosalpinx were recruited from the Center for Reproductive Medicine of the First Affiliated Hospital of Sun Yat-sen University, Guangzhou, China from April to December 2010. This study has been approved by the institutional review board of the First Affiliated Hospital of Sun Yat sen University in March, 2010 and informed consent was obtained from all participants.

All patients were aged below 40 years and had normal menstrual cycles. Endocrine examinations were normal and temperature monitoring revealed biphasic patterns. No patient had been administered with corticosteroids in the past 6 months. Exclusion criteria included endometriosis, uterine fibroids, polycystic ovary syndrome, ovarian tumor, unexplained infertility, immune infertility, chronic systemic diseases, sexually transmitted diseases, trophoblastic disease, smoking and alcohol consumption and male factor infertility.

### Diagnostic methods

Hysterosalpingography (HSG)was employed for hydrosalpinx diagnosis. Ultrasonography was carried out and it revealed bilateral or unilateral hydrosalpinx. HSG or laparoscopy (LAP) was employed to diagnose the tubal obstruction and ultrasonography did not reveal hydrosalpinx.

### Surgical treatment of hydrosalpinx

Under transvaginal ultrasound guidance, hydrosalpinx aspiration was performed; salpingostomy, proximal tubal ligation or salpingectomy were performed by laparoscopy.

### Sample collection and processing

Since day 10 of the menstrual cycle, urine LH test paper, transvaginal ultrasound and blood sex hormones were used to detect the LH peak. At approximately 7–8 days after ovulation, the endometrial tissue was collected at the fundus of the uterus and washed with normal saline to avoid contamination with blood. The samples were then fixed and embedded in paraffin, followed by sectioning. Secretory phase endometrium was pathologically confirmed. For patients with hydrosalpinx, sample collection was carried out before and after surgery during the implantation window, but sample collection was performed once in patients with tubal obstruction.

### Reagents for immunohistochemistry

Mouse anti-human LIF monoclonal antibody (1:80; R&D Systems, Minneapolis, MN, USA) and mouse anti-human L-selectin ligand monoclonal antibody (1:200; Santa Cruz Biotechnology, Inc., Santa Cruz, CA, USA) were used for immunohistochemistry which was carried out according to the manufacturer’s instructions.

### Determination

Five fields at high magnification (x400) were randomly selected from each section and Image-Pro Plus 5.1 software (Media Cybernetics, Shanghai, China) was employed for the detection of integrated optical density (IOD).

### Statistical analysis

Data are expressed as the means ± standard deviation. For patients with hydrosalpinx, data before and after surgery were compared using a paired t-test and those between hydrosalpinx patients and tubal obstruction patients were analyzed with an independent sample t-test. SPSS version 13.0 software for Windows was used for statistical analysis. A two-sided P-value of <0.05 was considered to indicate a statistically significant difference.

## Results

**Figure 1:**
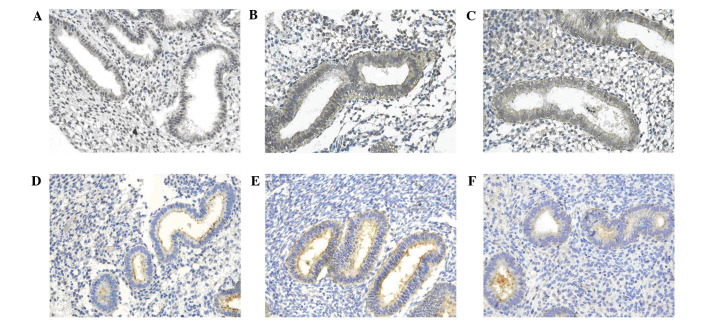
Immunohistochemistry staining showing leukemia inhibitory factor (LIF) and L-selectin ligand expression in the endometrium in hydrosalpinx patients (A and D) before surgery, (B and E) after surgery and (C and F) in tubal obstruction patients (magnification, x400). (A–C) LIF; (D–F) L-selectin ligand.

Under a light microscope, the expression of LIF and L-selectin ligand was mainly found in the cytoplasm and the cell membrane of endometrial epithelial cells, and less often found in the interstitium. LIF expression in the endometrium of hydrosalpinx patients was significantly different before and after surgery (P<0.05) (Table I). Before surgery, LIF expression in hydrosalpinx patients (Fig. 1A) was markedly different from that of tubal obstruction patients (Table II; Fig. 1C; P<0.05). After surgery, LIF expression in hydrosalpinx patients (Fig. 1B) was similar to that of tubal obstruction patients (P>0.05) (Table III). Before surgery, the integrin αvβ3 expression in hydrosalpinx patients (Fig. 1D) was markedly different from that of tubal obstruction patients (Table II; Fig. 1F; P<0.05). Following surgery, the integrin αvβ3 expression in hydrosalpinx patients (Fig. 1E) was comparable to that of tubal obstruction patients (P>0.05) (Table III).

## Discussion

LIF is a multifunctional factor belonging to the interleukin (IL)-6 family. The mature LIF is a secretory protein, highly glycosylated at asparagine residues and is composed of 180 amino acids. LIF binds to the corresponding receptors exerting biological effects, which may influence the reproductive activities at different levels, including follicular development, embryonic development, implantation and maintenance of pregnancy (5–7). LIF has been regarded as one of the markers for endometrial receptivity, having multiple biological activities. Studies have confirmed that LIF expression is present in ovaries, follicular fluid, fallopian tubes, trophoderm, endometrium and decidua.

LIF expression in the endometrium plays an important regulatory role in the blastocyst implantation in mammalians. LIF may strengthen the regulation and differentiation of the trophoderm, and plays an important role in the adhesion and invasion of blastocysts during implantation. LIF expression may be found in the natural killer cells in the decidua, chorion and endometrium during early pregnancy. There is evidence demonstrating that LIF regulates the blastocyst implantation via the regulation of the invasive ability of trophoblasts and by influencing the immune tolerance, and that LIF expression is required for blastocyst implantation (8–10). In the detection of mRNA species with the RNAase protection method, the results showed the protein and mRNA expression of LIF in the endometrial epithelial cells during the whole menstrual cycle. Notably, LIF expression is markedly increased in the endometrial epithelial cells during blastocyst implantation (middle and late secretory phase and early pregnancy) (11). Previous studies have shown that LIF expression in the endometrial epithelial cells rapidly increases during the middle and late luteal phase. However, during the middle and late luteal phase, LIF expression in patients with infertility from unknown causes was significantly lower than that in healthy subjects. In the middle luteal phase, LIF expression in the endometrium of patients with recurrent miscarriage was significantly reduced when compared with healthy child-bearing females (12). In addition, LIF expression in the endometrium in the secretory phase was 22-fold higher than that of the proliferative phase in females with a history of pregnancy, but LIF expression in the secretory phase is dramatically reduced in females with infertility (13). This suggests that LIF plays an important role in the initiation of blastocyst implantation and maintenance of pregnancy.

Our results showed that LIF expression in the endometrium of hydrosalpinx patients during the implantation window before surgery was markedly lower than that in the endometrium of tubal obstruction patients without hydrosalpinx. After surgery, LIF expression in the endometrium during the implantation window was comparable to that of the control group. In addition, LIF expression was significantly different before and after surgery. This indicates that hydrosalpinx influences LIF expression in the endometrium during the implantation window and that LIF expression increases after the surgical treatment of hydrosalpinx. These findings were consistent with those from previous studies. Seli *et al* (4) found that LIF expression in the endometrium of hydrosalpinx patients during the implantation window was markedly lower than that in the endometrium of healthy females, and that LIF expression increased after salpingectomy. These findings demonstrate that hydrosalpinx reduces LIF expression in the endometrium during the implantation window. Hydrosalpinx is a manifestation of chronic pelvic inflammation and the cytotoxic factors are at a high level in the affected fallopian tube (14). These factors might reflux into the uterus, influencing the endometrium. Copperman *et al* (15) compared the endometrium of hydrosalpinx patients and subjects with normal fallopian tubes. Their results showed that the number of inflammatory cells in the endometrium of hydrosalpinx patients was markedly higher than that in the endometrium of the controls, and that the expression of IL-2, a representative inflammatory cytokine, was also markedly increased in the endometrium of hydrosalpinx patients as compared with the controls. IL-2 is a specific cytokine secreted by the Th1 lymphocytes. Piccinni *et al* (16) found that Th1 cytokines can downregulate LIF expression. Thus, the increased IL-2 expression in the endometrium of hydrosalpinx patients may reduce LIF expression.

L-selectin is a member of the selectin family, which mediates the binding of white blood cells to endothelial cells and is involved in the migration of white blood cells through the vascular endothelial cells into inflammatory tissues, and the homing and recycling of lymphocytes (17,18). Previous studies have shown that reproduction, immunity and vascular functions have similarities at the molecular level (19,20).

The selectin family is a group of cellular adhesion molecules and consists of 3 molecules of similar structure: L-selectin, P-selectin and E-selectin, which were named as they were initially identified in the white blood cells, platelets and endothelial cells. To date, a total of 5 ligands of L-selectin have been identified: i) Glycosylation-dependent cell adhesion molecule-1 (GlyCAM-1) is a 50-kDa, secretory, salivary mucin that binds to selectins of more than 3 subtypes. The sulfated oligosaccharide chains of GlyCAM-1 bind to L-selectin. In addition, GlyCAM-1 binds to the lymphocytes, resulting in the activation of integrin β1 and β2; ii) CD34 is a 90-kDa glycoprotein and a type I transmembrane salivary mucin. CD34 is expressed in the endothelial cells of the vascular system, hematopoietic precursor cells, brain and embryonic fibroblasts; iii) mucosal addressin cell adhesion molecule-1 (MAdCAM-1) is a protein identified using MECA-367 monoclonal antibody. MAdCAM-1 is also a ligand of integrin α4β7 in lymphocytes; iv) Sgp200 is a sulphated glycoprotein which was identified in the separation of mouse high endothelial venules (HEVs) in the lymph nodes with chimera of L-selectin. Sgp200 is 200 kD in molecular weight and is secreted. The molecular characteristics of Sgp200 remain unclear; v) P-selectin glycoprotein ligand 1 also binds to L-selectin, exerting biological effects.

L-selectin is constitutively expressed in the majority of white blood cells. It mediates the adhesion between white blood cells and endothelial cells, and is involved in the migration of white blood cells through the endothelial cells into inflammatory tissues and the homing and recycling of lymphocytes. In addition, it mediates the adhesion between white blood cells. At the inflammatory sites, L-selectin initiates the adhesion between white blood cells and endothelial cells, which is mediated by the regulation of the expression of ligands on the endothelial cells. Genbacev *et al* (21) found that the migration of white blood cells across the blood vessels was morphologically similar to the adhesion of blastocysts to the uterus, and the blastocysts were in the liquid environment containing mucins secreted by the uterus during the adhesion of blastocysts to the uterus. Subsequently, Genbacev *et al* (21) found that the binding of L-selectin on the trophoblasts to its ligand could initiate the blastocyst implantation. During the implantation window, the expression of L-selectin on the blastocysts and that of its ligand in the endometrium have an increasing tendency. Their study not only confirmed the binding of L-selectin to its ligand, but it also demonstrated that L-selectin is an important regulator of pregnancy. Other studies have also revealed L-selectin expression in sperm, demonstrating its involvement in fertilization.

Ligands of L-selectin were first identified in mouse HEVs of lymph nodes, using MECA-79 monoclonal antibody. Thus the corresponding antigen was named peripheral lymph node addressin (PNAd) which includes G1yCAM-1, CD34 and Sgp200, for example.

In a previous study, the antibody against the ligand of L-selectin was used in the immunofluorescence detection of endometrium in the follicular and luteal phase (22). The results showed that the endometrium was weakly positive for MECA-79 in the follicular phase and the MECA-79 positive cells were found in the endometrial glands and epithelial cells of the uterine cavity. In the luteal phase, the endometrium was markedly positive for MECA-79, especially in cells of the uterine cavity. Continuous biopsy of the endometrium and subsequent western blot assay for MECA-79 showed that MECA-79 expression on days 3 and 6 was markedly increased when compared with that on days 0 and 2. This suggested that the endometrium became receptive and the expression of the ligand on L-selectin increased. In females with normal menstrual cycles, MECA-79 expression in the endometrium has a similar tendency.

To date, few studies have been conducted to investigate L-selectin and its ligands in the endometrium and little is known regarding the effect of hydrosalpinx on the expression of L-selectin and its ligands.

In the present study, L-selectin ligand expression in the endometrium during the implantation window in hydrosalpinx patients before surgery was markedly lower than that of patients with tubal obstruction. After surgery, L-selectin ligand expression during the implantation window in hydrosalpinx patients was comparable to that of tubal obstruction patients. In addition, L-selectin ligand expression was significantly different before and after surgery. This suggests that hydrosalpinx influences the L-selectin ligand expression in the endometrium during the implantation window and that L-selectin ligand expression increases after surgery.

Hydrosalpinx is a manifestation of chronic pelvic inflammation. Copperman *et al* (15) compared the endometrium of hydrosalpinx patients and subjects with normal fallopian tubes. Their results demonstrated that the number of inflammatory cells in the endometrium of hydrosalpinx patients was markedly higher than that of the controls, and the expression of IL-2, a representative inflammatory cytokine, was also significantly increased in the endometrium of hydrosalpinx patients as compared with controls. IL-2 is a specific cytokine secreted by the Th1 lymphocytes. Piccinni *et al* (16) found that Th1 cytokines can downregulate LIF expression. Thus, the increased IL-2 expression in the endometriun of hydrosalpinx patients may reduce the LIF expression. The cytotoxic factors in the affected fallopian tube were at a high level (14). These factors might reflux into the uterus, influencing the L-selectin ligand expression in the endometrium. Findings in the present study and previous studies demonstrated that hydrosalpinx could reduce the expression of well-known markers for endometrial receptivity, including LIF. Our findings also revealed hydrosalpinx reduced the expression of L-selectin ligand in the endometrium. On the basis that the change in LIF expression was consistent with that of L-selectin ligand in the endometrium of hydrosalpinx patients, we postulated that L-selectin and its ligand could be used as markers for endometrial receptivity. However, more multicenter, prospective, randomized controlled studies with large sample sizes would be required to confirm our findings.

Overall, the endometrium undergoes cyclic regeneration that varies in different individuals. Accurate evaluation of endometrial receptivity is the basis for the improvement of endometrial receptivity. In the present study, the effect of hydrosalpinx on the expression of LIF and L-selectin ligand in the endometrium was investigated. Our findings revealed that hydrosalpinx influenced the expression of LIF and L-selectin ligand in the endometrium, which compromised the endome-trial receptivity of blastocyst implantation and the ability to maintain pregnancy. The expression of LIF and L-selectin ligand in the endometrium was increased after surgical intervention of hydrosalpinx.

## Figures and Tables

**Table I:** Expression of LIF and L-selectin ligand in hydrosalpinx patients before and after surgery.

	Before surgery (n=60)	After surgery (n=60)	P-value
LIF	0.42±0.17	0.58±0.21	<0.05
L-selectin ligand	0.37±0.11	0.54±0.15	<0.05

LIF, leukemia inhibitory factor.

**Table II:** Expression of LIF and L-selectin ligand in hydrosalpinx patients before surgery and in tubal obstruction patients.

	Before surgery (n=60)	Control group (n=30)	P-value
LIF	0.42±0.17	0.60±022	0.000
L-selectin ligand	0.37±0.11	0.50±0.15	0.000

LIF, leukemia inhibitory factor.

**Table III:** Expression of LIF and L-selectin ligand in hydrosalpinx patients after surgery and in tubal obstruction patients.

	After surgery (n=60)	Control group (n=30)	P-value
LIF	0.58±0.21	0.60±022	0.662
L-selectin ligand	0.54±0.15	0.50±0.15	0.302

LIF, leukemia inhibitory factor.
